# Barriers to effective clinical supervision from the perspective of nurses: A descriptive qualitative study

**DOI:** 10.1002/nop2.2028

**Published:** 2023-12-14

**Authors:** Vajihe Atashi, Maryam Movahedi Najafabadi, Atefeh Afshari, Somayeh Ghafari

**Affiliations:** ^1^ Nursing and Midwifery Care Research Center, Adult Health Nursing Departement Isfahan University of Medical Sciences Isfahan Iran; ^2^ Trauma Nursing Research Center Kashan University of Medical Sciences Kashan Iran; ^3^ Nursing and Midwifery Care Research Center, Department of Community Health Nursing, Faculty of Nursing and Midwifery Isfahan University of Medical Sciences Isfahan Iran; ^4^ Nursing and Midwifery Care Research Center, Department of Critical Care Nursing, Faculty of Nursing and Midwifery Isfahan University of Medical Sciences Isfahan Iran

**Keywords:** barriers, effective clinical supervision, nurses, qualitative study

## Abstract

**Aim:**

The present study aimed to explain the barriers to effective clinical supervision from the perspective of nurses.

**Design:**

Qualitative descriptive study.

**Methods:**

The present study enrolled 21 nurses selected by purposive sampling. Data were collected through semi‐structured interviews, which were digitally recorded and immediately transcribed verbatim, and analysed using content analysis method.

**Results:**

From the nurses' perspective, the influential barriers included poor academic, ethical, communicational, professional competencies at the passive management level, defects in supervision prerequisites, conventional beliefs, ineffective organization, shortage of workforce at the level of inappropriate context, lack of motivation and poor accountability at inadequate professional maturity level.

**Public Contribution:**

The following items affect how clinical supervision is implemented: Motivation, accountability at the personal level, sufficient workforce, conducive conditions, effective organization of resources, and preparing the individual for supervision at the organizational level to implement effective clinical supervision.

## 1 INTRODUCTION

Supervision is one of the key principles of management and an important pillar of modern management in all organizations (Ogbeide et al., 2022). This pillar is so important and necessary in management that it can ensure all elements move in the right direction to achieve predetermined goals (Sewell et al., 2023).

The need for and importance of control and supervision are highlighted in the healthcare organizations because they pursue many important goals such as preserving human health and life, providing quality services, and meeting patient needs, which are more complex than the needs in any other organization (Snowdon et al., 2020).

To transform and improve services in the unit under their supervision, nurses need to pay particular attention to the supervision and control of nursing services (Akter et al., 2019).

Clinical supervision has been recognized as an inseparable part of the function and an important and superior element of nursing development (Milne & Reiser, 2023), and it is an educational process aiming to promote, mature, and develop the individual being supervised (Setyowati et al., 2023). Clinical supervision is one of the core activities in care professions and is considered the main route for professional support and progress of nurses (Driscoll et al., 2019). It is also recognized as the second out of the three main leadership behaviours. It has also been proposed as a tool for ensuring the quality of care (Inocian et al., 2022). Clinical supervision is one of the supervisory techniques, regarded as a mechanism for improving nurses' professional knowledge and skills, and the effectiveness of practice and enforcing standards (Gershuni et al., 2023). In order to receive the supervisor's support and share experiences with other nurses familiar with particular professional problems (Mohamed & Ahmed, 2019). However, it has occasionally been considered a difficult and threatening test that entails disciplinary actions for personnel (Schmitt et al., 2023). Clinical supervision has been discussed among nurses, academics, clinicians, managers, and policy‐makers since the early 1990's, and such debates and published articles have affected the acceptance of clinical supervision throughout the nursing profession (Ogbeide et al., 2022). Clinical supervision is increasingly recognized as the key to ensuring safe nursing practice. Furthermore, greater attention is now being paid to supervision due to the transformed nature of nursing practice and distancing from duty‐based nursing toward a holistic approach to care and the importance of nurse–patient communications based on empathy, honesty, mutual respect, and participation (Reynolds & McLean, 2023).

Clinical supervision is a process in which a nurse's clinical practice is constantly monitored and assessed by another person, and receives feedback, which leads to the development of her professional skills (Hamilton et al., 2023), or a systematic and ongoing process through which any supervised person can review her performance and develop and maintain her care skills, knowledge and standards. As such, it provides a mechanism for ongoing professional development, and identifies and supports personal and professional learning needs (Driscoll et al., 2019).

Clinical supervision provides an opportunity for identifying good practices, effective communications, increased interdisciplinary teamwork, respectful and reassuring relationships with colleagues, sharing beliefs with colleagues, receiving feedback and facilitating nursing reviews on care problems (Purfarzad et al., 2019). Effective clinical supervision also has positive outcomes for the patients and leads to identifying and meeting care needs, improving the quality of care, improving care standards, better patient outcomes, a greater focus on patient–nurse interaction, and using effective strategies for nurse–patient communications (Inocian et al., 2022). Although the benefits of clinical supervision have not been scrutinized in articles (Purfarzad et al., 2019), and its many positive outcomes have been raised, there is a need for proper understanding of barriers to implementation of effective clinical supervision before such benefits can be realized (Milne & Reiser, 2023). Despite significant development and appreciation of the value of clinical supervision, the knowledge about barriers to effective clinical supervision still remains inadequate (Hamilton et al., 2023). Moreover, the barriers to effective clinical supervision in healthcare systems are still not fully known because few studies have examined them (Ogbeide et al., 2022). Meanwhile, there is a need for a more profound understanding of barriers to the process of effective clinical supervision (Masamha et al., 2022). On the other hand, what is controversial is the context of Iranian hospitals. Empirical evidence suggests that the benefits claimed in articles are not as such in reality. For example, a study by Posluns & Gall showed that clinical supervision led to increased work pressure, stress, and disheartenment in nurses (Posluns & Gall, 2020).

As barriers to effective clinical supervision in Iranian culture are not very clear, and previous studies have been quantitative, which often include limited number of variables and cannot explain people's views, thoughts, and perceptions, or show effective factors, qualitative studies can fill this gap and provide a more accurate insight into this subject (Speziale et al., 2011). Thus, the present study was conducted to investigate barriers to effective clinical supervision from the nurses' perspective, such that nurses' experiences in this respect can be identified. Once the barriers affecting implementation of preventive measures are identified, useful information can be provided for the nursing policy‐makers, planners, and managers to eliminate barriers to effective clinical supervision.

## 2 STUDY METHOD AND SAMPLES

We used a descriptive–qualitative method. Descriptive–qualitative studies are used for describing and exploring the depth and complexities of a phenomenon, problem, or a subject. This kind of research can provide answers to specific questions relating to clinical personnel and policy‐makers such as individuals' concerns about an event, individuals' responses (experiences, knowledge, attitude, feelings, and views) to an event, and its facilitators and barriers (Sandelowski, 2000). The samples included 21 nurses selected by purposive sampling with maximum diversity in terms of work experience, position, age, gender, and education. The participants worked in different clinical roles, including nurse, head nurse, and supervisor. In purposive sampling, researchers deliberately select their participants based on two criteria: First, compatibility of experience with research question, and second, having the attributes of a “good informant”. The inclusion criteria included supervisors, head‐nurses or nurses working in intensive care unit, internal surgery, and emergency department willing to participate and share their experiences (Table 1).

**TABLE 1:** Study participants' characteristics.

Variable	*N*	%
Age		
20–30	6	28.58
31–40	10	47.62
41–50	5	23.80
Educational degree		
Bachelor's	17	80.95
Master's	4	19.05
Professional experience		
Less than 1 year	4	19.05
1–5 years	5	23.80
More than 5 years	12	57.15
Official position		
Nurse	11	52.40
Head nurse	5	23.80
Supervisor	5	23.80

### 2.1 Study setting

The present study was conducted in a teaching hospital affiliated with (REDACTED name) University of Medical Sciences. This hospital has 267 active beds in pulmonary, urology, gastroenterology, emergency, CCU, ICU, and haemodialysis departments. The nurse‐to‐bed ratio is 1:6 in internal wards and 1:2 in ICU/CCU in the morning shift, and 1:10 and 1:2.5, respectively, in the evening and night shifts. There are a training supervisor, an infection control nurse, and two clinical supervisors in the morning shift, which reduces to just one clinical supervisor in the afternoon and night shifts.

### 2.2 Ethical considerations

First the project was approved by necessary permissions were obtained from the officials and Ethics Committee of the university. After being introduced to hospital authorities, the participants were briefed on the study objectives, and asked for permission to record their voices. The participants were assured of confidentiality of data and being free to withdraw from the study at any stage before signing the informed consent form.

### 2.3 Data collection

Data were collected through semi‐structured interviews from May to December 2019. To conduct the interviews, first, a list of guide questions was prepared based on the study question and review of relevant literature. The interview guide included semi‐structured questions relating to barriers to effective clinical supervision. To ensure its content validity, the interview guide was assessed by two nursing faculty members with expertise in qualitative studies, and a supervisor with a Bachelor's degree in nursing with 12 years of work experience. Then, the interview guide was assessed in two pilot interviews. The interviews were assessed by the second author, but no change was made in the interview guide questions. First, interviews began with a general question: What is effective clinical supervision? Interviews were conducted individually in the participants' office or one of the rooms in ICU/CCU in Persian. The place and time of interviews were decided by the participants. All the interviews were conducted by the principle researcher. Each interview lasted 30–90 min depending on the participants' willingness. During the interview, the participants were encouraged to share their experiences of barriers to effective clinical supervision. For example, they were asked: What are the attributes of effective clinical supervision? What factors affect effective clinical supervision? Which are the barriers to effective clinical supervision in your view? The interview continued with further probing questions to obtain deeper information and clarify the concept. The participants were asked to clarify the concepts through objective examples. Moreover, the following questions were used to deepen the interview: “What do you mean?”, “Please elaborate on that.”, “This is my understanding of your words, is that right?”. The interviews were recorded, and then immediately transcribed verbatim. It should be noted that two of the participants required more time to ponder on the researcher's questions. Hence, there was a second interview with them. Sampling continued until previous data were repeated and data saturation was reached.

### 2.4 Data analysis

Qualitative content analysis is highly useful for many studies in the field of health, especially when they are descriptive, or intend to investigate a particular issue in a specific group of people. In the present study, Graneheim & Lundman method of qualitative content analysis was used to analyse the data with an inferential approach (Graneheim & Lundman, 2004). Data were analysed as they were collected, and all recorded interviews were first transcribed verbatim within 48 h by the first author. Then, to be fully engaged in the data and develop a general feeling about them, two authors carefully listened to the first two interviews and reviewed the transcripts several times, and independently selected the meaning units and summarized, and discussed them to resolve any disagreements. Next, the first author analysed the remaining 21 interviews as follows: first, she listened to the audio files and read their transcript as well as the field notes several times to develop a complete understanding. Then, the entire text was divided into smaller parts in a table, and each was referred to as a meaning unit. The general concept of each of the summarized meaning units was inserted into a column called condensed meaning unit. In the final stage, the entire transcript transformed into codes through encoding the concepts of condensed meaning unit column. Then, codes were categorized according to their differences and similarities, and a title was given to each category, which encompassed all codes in that category. Finally, these categories were assigned to larger categories as much as possible (subcategories) with the aim to obtain new knowledge, improve understanding and describe barriers to effective supervision. Subcategories that were rather similar were combined, from which, the main categories emerged, and ultimately the main theme.

### 2.5 Study rigor

The following four criteria were used to confirm the study rigor: Conformability, transferability, credibility, and dependability.

### 2.6 Conformability

This was done through audit trails, which means that the researcher accurately recorded and reported all stages and processes of the study to enable others to follow up.

### 2.7 Credibility

This was confirmed through sampling with maximum diversity, prolonged engagement of the researcher with the study setting, peer debriefing, and participants' assessment of the results.

### 2.8 Dependability

This was determined through external check, such that parts of the transcripts, relevant codes, and categories were sent to two observers familiar with qualitative research to be assessed and confirmed.

### 2.9 Transferability

The use of demographically different participants in terms of age, experience and employment status, and providing accurate details of the subject helped transferability of the results.

## 3 RESULTS

Analysis of the interview data led to the identification of 11 subcategories, three main categories, and a theme, which reflected the nurses' perception of barriers to effective clinical supervision (Table 2).

**TABLE 2:** Study results (codes, subcategories, main category, theme).

Code	Subcategory	Category	Theme
Lack of knowledge	Lack of academic competency	Passive management	Supervision in ineffective context
Lack of clinical skill			
Low accountability	Lack of ethical competency		
Poor commitment			
Supervision just to meet requirements			
Poor support	Lack of professional competency		
Poor decisiveness			
Not being a role model			
Not being an encourager			
No being an active listener	Lack of communicational competency		
Lack of mutual trust between the supervisor and the supervised			
Improper conduct			
Disrespect for people's integrity			
Unclear job description	Lack of supervision prerequisites	Inappropriate context	
Huge workload			
Number of duties			
Lack of useful training courses			
Ineffective system of introducing rules			
Shortage of nurses	Shortage of workforce		
Shortage of supervisors			
Lack of sufficient authority	Ineffective organization		
Lack of unity of direction			
Lack of continuity in supervision			
Subjectivity of supervision process			
Generalized supervision forms			
Supervision unimportant to senior managers	Conventional beliefs and attitudes		
Neglecting meritocracy			
Individual‐orientation			
Doctor's dominance			
Prioritizing other duties over supervision			
Perceived discrimination by nurses	Lack of job motivation	Professional immaturity	
Managers' improper perception of working conditions			
Nurses disheartenment with management system			
Lack of feeling responsible	Poor accountability		
Routine care			

### 3.1 Passive management

Passive management was one of the main categories. What is meant by passive management is the lack of personal and professional attributes that enable the manager to supervise and have a considerable impact on the effective supervision, which included the following four subcategories: poor academic competency, poor ethical competency, poor professional competency, and poor communicational competency.

### 3.2 Poor academic competency

The participants believed that the supervisor's lack of clinical knowledge and experience is one of the main barriers to effective clinical supervision. In this respect, one of the participants commented:

Some supervisors are totally unknowledgeable. They have no knowledge. For instance, they have no information about our ward, which is an intensive ward. They don't know how to provide care for a patient under ventilation. How are they supposed to supervise? (P21).

### 3.3 Poor ethical competency

The supervisors' lack of ethical competency was another barrier o effective clinical supervision. Ethical competency is a way of improving the quality of nursing services. In view of the participants, it included supervision for the sake of duty, poor accountability, and not being a role‐model in terms of work and performance.

One of the supervisors is too carefree in her work, or gives in easily, and this gives the personnel an excuse to obey her less often. For instance, she chitchats with some personnel, or drinks tea with them, so she can no longer ask them not to eat and drink in the station or to provide proper patient care (P5).

### 3.4 Poor professional competency

Professional competency is another attribute of a clinical supervisor. It means the effectiveness and ability of the supervisor in leading the hospital and personnel to achieve patient and personnel satisfaction using available means. A participant stated:

Knowledge of us supervisors is important, especially managerial knowledge. How to make a decision and what criteria we should use are very important in our work (P11).

### 3.5 Poor communicational competency

The participants believed that good communication and appropriate treatment are prerequisite for a clinical supervisor. When supervisors are efficient, polite, and affable, discuss issues in good manners, treat the supervised like a friend, and have a good demeanor such that the supervised feels comfortable with the supervisor and not her superiority, they will be very much accepted. In this respect, one of the participants said:

One of the supervisors greets us when they arrive. Some supervisors think the personnel are their servants, and look down on us even in supervising activities, and they are not with us, but against us (P6).

### 3.6 Inappropriate context

This refers to factors associated with supervision infrastructure and human resources management. This main category included four subcategories: defects in supervision prerequisites, workforce shortage, ineffective organization in supervision, and conventional beliefs and attitudes.

### 3.7 Workforce shortage

Human resources are among the main conditions for effective clinical supervision, as was cited by almost all the participants. Shortage of supervisors and nurses is one of the main barriers to the implementation of effective clinical supervision. One of the participants explained:

There are too many wards and only a few supervisors. That is why they just pass by. But I think they are right not to supervise properly because there are only few of them and workload is huge (P12).

Another participant said:

With only few nurses, we are forced to overlook certain errors because if we asked them to go to another ward because of shortage of workforce, they resist or won't go. Shortage of workforce practically affects our supervising task, and personnel take advantage of this (P3).

### 3.8 Defects in supervision prerequisites

High‐quality supervision cannot be expected when the supervision infrastructure is not in place or workforce is not properly trained, and rules and regulations and job description are not properly written. One of the supervisors revealed:

Our role is coordination rather than supervision. We have to carry out the job of the guard and the receptionist, and don't know what our duties are. I have to arrange for the ambulance to transfer the patient to another hospital, and many other tasks unrelated to clinical supervision (P4).

Supervision requires training. As a supervisor, I have to be trained. They have to hold educational classes on supervision for us. We have not passed any management courses, and don't know what to do or how to react in a particular situation (P18).

### 3.9 Inefficient organization

This includes inadequate authority, lack of unity of direction in supervising method, discontinuity in supervision, and subjectivity of supervising forms. One of the participants said:

One of the problems is waking in the morning after a night shift. I ask where the personnel are, but some supervisors wouldn't ask, and this causes discoordination among supervisors, and the personnel tell me that I am doing wrong (P2).

Some participants stated that clinical supervision is not efficient enough to assess the performance of staff. One of the participants commented:

Clinical supervision isn't objective enough, and performance of personnel cannot be assessed in this way (P1).

### 3.10 Conventional beliefs and attitudes

According to participants, conventional beliefs and attitudes were another barrier to effective clinical supervision, and included the beliefs such as individual‐orientation, physician‐orientation, lack of attention to meritocracy, and senior managers' negligence of supervision. In this respect, one of the participants said:

I may see an error during supervision and show not reaction because the person is one of the manager's associates (P13).

The idea of doctors' dominance has made non‐physician groups lose their motivation for service. One of the participants said:

Some of the problems of supervision have to do with top management and doctors' dominance. All our supervising officials are doctors, and although we have matrons, doctors have the last word (P10).

### 3.11 Professional immaturity

This main category included two subcategories: Poor‐occupational motivation and poor accountability.

### 3.12 Poor occupational motivation

As stated by the participants, another barrier to clinical supervision was the motivation. Accordingly, huge workload, managers' improper understanding of working conditions, perceived discrimination, and disheartenment have affected nurses' motivation, prevented proper implementation of clinical supervision, and achieving high quality of care. A participant explained:

In nursing, I have to attend all morning, afternoon, and night shifts, for what income? And with the exhaustion we experience in these shifts, what motivation is left for us to perform quality care? Yet, they supervise and criticize us (P19).

### 3.13 Poor accountability

According to the participants' experience, accountability is an inner feeling of obligation and commitment, which nurses believe in and are committed to. In other words, it is being conscientious. One of the participants said:

Most tasks are done routinely and at a specific time. This shouldn't have happened if clinical supervision was good. Some colleagues even register the tasks they haven't done just to avoid being criticized by the supervisor (P3).

The main categories of passive management, inappropriate context, and professional immaturity formed a larger and more abstract theme of supervision in an ineffective context, which was identified the main study theme.

## 4 DISCUSSION

The present study was conducted to explain barriers to effective clinical supervision from the perspective of nurses. The results showed that these barriers include passive management, inappropriate context, and professional immaturity. The codes obtained are discussed below.

### 4.1 Passive management

According to the results, an efficient supervisor should possess a wide range of academic, ethical, communicational, and professional attributes, and shortfall in any of these components creates a barrier to effective supervision (Dawes & Topp, 2022). One of the categories was poor academic competency. Based on the participants' experiences, a supervisor should first have better clinical knowledge and skill than the personnel to be able to supervise their care and healthcare performance. They should know standards of care and procedures well, and update themselves. Otherwise, precise and comprehensive supervision of the supervised will not be possible. A study conducted by Gershuni et al showed that clinical supervisors lack the necessary knowledge and competency for supervision (Gershuni et al., 2023). Tay et al also believe that clinical supervision over nurses' performance is a role of high responsibility, which requires formal training to strengthen skills, knowledge, and awareness in addition to experience (Tay et al., 2023).

Another finding was poor‐ethical competency of supervisors. According to the participants' narratives, supervisors should take into account ethical norms such as accountability and commitment. In a study by Sumner, Fradet, & Burguete, researchers argued that a supervisor's role depends on preparedness, knowledge, and accountability, and that accountability is one of the necessary and vital attributes for performing a supervisory role (Sumner et al., 2023).

Professional competency is one of the basic principles for supervisors, and an efficient supervisor should have professional knowledge, be decisive, supportive, encouraging, a role model, and a feedbacker. According to the results obtained by Parry, the supervised can only accept and take note of the supervisor when they feel the supervisor ensures quality of care and supports them (Parry, 2023). However, Caputo et al. stated that nursing managers have not been empowered for managerial and supervisory roles, and for providing leadership and supportive behaviours needed by nurses (Caputo et al., 2023). Vandette & Gosselin regard clinical supervision as a kind of supportive strategy that improves creativity and organizational atmosphere and may be a buffer for interpersonal problems (Vandette & Gosselin, 2019).

Another group of attributes needed for effective clinical supervision is communicational competency. According to Lee & Thackeray, since the nature of the clinical supervision process is interactive and dynamic, the relationship between the supervisor and the supervised has important effects on clinical supervision, and the better the relationship is, the better and more effective the clinical supervision will be (Lee & Thackeray, 2023). Schmutz et al. (2019) also reported that trust and mutual interactive relationships are effective in clinical supervision (Schmutz et al., 2019).

### 4.2 Inappropriate context

Some of the participants argued that an inappropriate context such as shortage of workforce, defects in supervision prerequisites, inefficient organization, and conventional beliefs and attitudes are among important barriers to effective clinical supervision.

According to the participants' experiences, factors such as lack of time due to huge workload and diversity of activities affect supervision, and reduce its effectiveness. Young, McEntee, & Bennett stated that absence of specialized supervision is one of the factors resulting in ineffective clinical supervision (Young et al., 2023).

Ogbeide et al. reported that the absence of unity of direction in supervision when individuals enforce their own tastes disrupts effective clinical supervision (Ogbeide et al., 2022). Hill & Abhayasinghe mentioned that the supervisors' lack of clear guidelines for performing their roles is a barrier to successful clinical supervision (Hill & Abhayasinghe, 2022). Moreover, Howard et al. argued that lack of a working model and method for supervisors is among factors for ineffective clinical supervision (Howard et al., 2023).

The present study participants considered conventional beliefs and attitudes as another group of barriers to implementation of effective clinical supervision. The lack of clinical supervision for senior managers, individual‐orientation, doctors' dominance, and neglecting meritocracy fall in this important category. The effect of rules and regulations diminishes when working relationships are based on individual‐orientation or doctors' dominance. Also, clinical supervision become less effective when senior managers regard it as low priority compared to other duties. The results obtained by Saab et al. showed that attitudes such as doctors' dominance and lack of attention to meritocracy in the healthcare system are among important barriers to effective clinical supervision (Saab et al., 2021).

### 4.3 Professional immaturity

From nurses' perspective, another barrier to effective clinical supervision was the professional immaturity of the supervised. This category included poor‐job motivation and poor accountability.

Lack of motivation was another finding in the category of poor personal competency. The present study results showed that the supervised perceived discrimination, managers' inappropriate perception of the nurses' difficult working conditions, and disheartenment were among causes of nurses' low motivation, which per se led to worthlessness of the supervision process for them. Thapa et al. found that nurses' lack of motivation was one of the barriers to performing professional roles (Thapa et al., 2022).

According to the participants, nurses' poor accountability was another barrier to effective clinical supervision. This category included providing routine care instead of proper care, and poor accountability. The results showed that nurses attached the most importance to routine care, and carried out their duties to the extent that they would not be questioned. Shahzeydi et al. stated that routine centeredness of nursing practice is regarded as a serious defect in nursing. (Shahzeydi et al., 2022).

## 5 CONCLUSION

The present study results explained barriers to effective clinical supervision from nurses' perspective. According to the results, barriers to effective clinical supervision are diverse and complex, and associated with personal and organizational levels. Their motivation and skills in different fields at personal level, sufficient workforce, appropriate conditions, effective organization of resources, and preparation of the individual for supervision at organizational level are like interdependent loops that affect implementation of effective clinical supervision. Hence, the working context is important for achieving the objectives of clinical supervision such as improved quality of care, improved care standards, progress of safe nursing care, and professional development of nurses, such that clinical supervision objectives cannot be achieved if the working context is not reformed. Furthermore, changes happen faster in the personnel's attributes in a structured working environment.

### 5.1 What does this article contribute to the wider global clinical community?

- The results of this study provide an insight into the barriers to effective clinical supervision from the nurses' perspective. Once the barriers affecting implementation of preventive measures are identified, useful information can be provided for the nursing policy‐makers, planners, and managers to eliminate barriers to effective clinical supervision.
- Researchers and decision‐makers in the area of healthcare delivery in other countries can also use the findings of the present study to identify the barriers and intervention areas for effective clinical supervision based on the immediate sociocultural, political, and financial context.

## FUNDING INFORMATION

This study was financially supported by the Research Administration of Isfahan University of Medical Sciences (project number: 297093, IR.MUI.RESEARCH.REC.1397.307).

## CONFLICT OF INTEREST STATEMENT

The authors declared no conflict of interests.

## ETHICS STATEMENT

The Ethics Committee of University of Medical Sciences, Iran, approved this study (code: 1397.307). All participants provided verbal consent for participation. Moreover, we obtained their verbal consent for recording the interviews and group discussions and ensured them of the anonymous handling and reporting of their information.

The paper has been submitted with full responsibility, following due ethical procedure, and there is no duplicate publication, fraud, plagiarism.

## Data Availability

None.
